# Role of early minimal-invasive spine fixation in acute thoracic and lumbar spine trauma

**DOI:** 10.4103/0019-5413.37003

**Published:** 2007

**Authors:** Oliver I Schmidt, Sergej Strasser, Victoria Kaufmann, Ewald Strasser, Ralf H Gahr

**Affiliations:** Trauma Centre, St.Georg, Klinikum Sankt Georg, Delitzscher Strasse 141, 04129 Leipzig, Germany; * Department of Neurology, Klinikum Sankt Georg, Delitzscher Strasse 141, 04129 Leipzig, Germany

**Keywords:** Damage control, minimally invasive, polytrauma, spinal fracture, spinal instrumentation

## Abstract

Polytraumatized patients following a severe trauma suffer from substantial disturbances of the immune system. Secondary organ dysfunction syndromes due to early hyperinflammation and late immunparalysis contribute to adverse outcome. Consequently the principle of damage control surgery / orthopedics developed in the last two decades to limit secondary iatrogenic insult in these patients. New percutaneous internal fixators provide implants for a damage control approach of spinal trauma in polytraumatized patients. The goal of this study is to evaluate the feasibility of minimal-invasive instrumentation in the setting of minor and major trauma and to discuss the potential benefits and drawbacks of this procedure.

**Materials and Methods:**

The present study is a prospective analysis of 76 consecutive patients (mean age 53.3 years) with thoracolumbar spine fractures following major or minor trauma from August 2003 to January 2007 who were subjected to minimal-invasive dorsal instrumentation using CD Horizon^®^ Sextant™ Rod Insertion System and Longitude™ Rod Insertion System (Medtronic^®^ Sofamor Danek). Perioperative and postoperative outcome measures including e.g. local and systemic complications were assessed and discussed.

**Results:**

Forty-nine patients (64.5%) suffered from minor trauma (Injury Severity Score <16). Polytraumatized patients (n=27; 35.5%) had associated chest (n=20) and traumatic brain injuries (n=22). For mono- and bisegmental dorsal instrumentation the Sextant™ was used in 60 patients, whereas in 16 longer ranging instrumentations the (prototype) Longitude™ system was implanted. Operation time was substantially lower than in conventional approach at minimum 22.5 min for Sextant and 36.2 min for Longitude™, respectively. Geriatric patients with high perioperative risk according to ASA classification benefited from the less invasive approach and lack of approach-related complications including no substantial blood loss.

**Conclusion:**

Low rate of approach-related complications in association with short operation time and virtually no blood loss is beneficial in the setting of polytraumatized patients regarding damage control orthopedics, as well as in geriatric patients with high perioperative risk. The minimal-invasive instrumentation of the spine is associated with beneficial outcome in a selected patient population.

Fractures of the thoracolumbar spine have been stabilized using dorsal instrumentation for more than three decades. The main disadvantage of dorsal instrumentation is the need for a large midline incision and significant paraspinal muscle dissection. Flexion-distraction injury, classified as B-Type fractures in the European,^1^ develop substantial secondary kyphosis due to the lack of active tension banding from paraspinous muscles and posterior ligament complex (PLC). The conventional exposure of entry points for pedicle screws during dorsal instrumentation procedures produces substantial iatrogenic damage associated with extensive blood loss, prolonged hospitalization and significant cost.^2^

Different types of minimal-invasive systems for dorsal instrumentation have been described so far,^3^–^5^ but no implant or procedure has been widely accepted. Originally designed by a neurosurgeon specialized in degenerative spine diseases,^6^ the CD Horizon^®^ Sextant™ Rod Insertion System (Medtronic^®^ Sofamor Danek) is a percutaneous dorsal fixation system of polyaxial cannulated pedicle screws, curved rods and a targeting device, resembling a nautical sextant, thus originating the nomenclature. In addition, further development of Sextant™ created the Longitude™ System that features the same screws with a free-hand rod inserter for longer ranging instrumentations.

In 2003 we started using the CD Horizon^®^ Sextant™ Rod Insertion System for trauma patients, who suffered from flexion-distraction fractures without neurological deficit. These patients did not need dorsal decompression; hence no conventional open approach to the spine was necessary. Using the percutaneous system as a tension band device^7^,^8^ it reduced iatrogenic damage to the PLC and paraspinous muscles in these patients. Virtually no blood loss was documented and patients recovered quickly from the surgical procedure. The operating time also was substantially reduced in comparison to conventional open approach. Therefore, indication for minimal-invasive instrumentation was expanded to first day surgery in polytraumatized patients. In matters of immunologic disturbances following polytrauma, these patients benefit from short operating time and low iatrogenic antigen load, according to the recently evolving principle of damage control surgery/ orthopedics.^9^,^10^ A trend towards reduced pulmonary and ICU-related complications is also demonstrated.^11^

Vertebral compression fractures (Type A),^1^ are normally prescribed bracing. Many patients prefer surgery in these controversial cases to avoid bracing, achieve full motion and consequently quick return to work. The geriatric patients who suffer from osteoporotic fractures have a degenerated spine with previously existing spinal canal compromise. A combination of vertebroplasty and minimal-invasive instrumentation has been successfully performed routinely to prevent secondary adjacent fractures and further deterioration of spinal canal space.

The goal of this study is to evaluate the feasibility of minimal-invasive dorsal instrumentation using the Sextant™ and Longitude™ implants in percutaneous stabilization procedures of the spine in the setting of minor and major trauma. This is the first study to be conducted to evaluate the potential benefits and drawbacks of these implants

## MATERIALS AND METHODS

**Figure 1:**
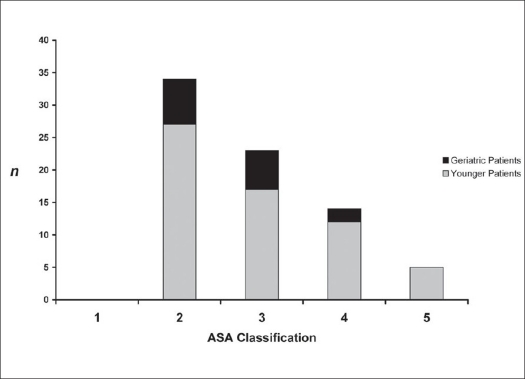
Preoperative physical fitness according to ASA classification, demonstrating low physical fitness prior to surgery in the investigated geriatric patient population (aged 70 and above). All polytraumatized patients were found in the younger patient group indicated by ASA Class 4 and 5. (n = patient number; ASA classification, 1 = Class 1; 2 = Class 2; 3 = Class 3; 4 = Class 4; 5 = Class 5)

**Figure 2:**
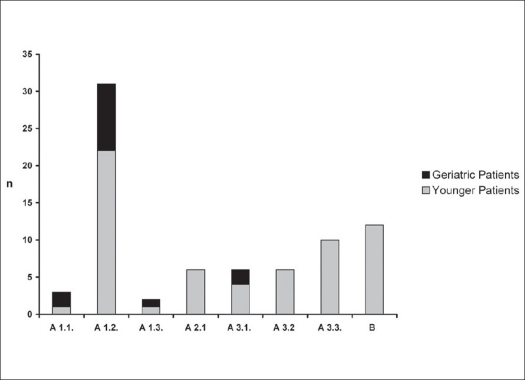
Classification of thoracolumbar fractures according to Magerl et al reveals stable anterior column fracture Type A 1.2 to be the most frequent stabilized by minimal invasive percutaneous dorsal instrumentation. More severe spine fractures in polytraumatized patients are seen in the younger population group only (n = patient number; A 1 = compression injury of the endplate; A 2 = compression injury and split fracture; A 3 = complete burst fracture; B = flexiondistraction injury)

Seventy-six consecutive patients of thoracolumbar fractures were stabilized by percutaneous dorsal stabilizations using either CD Horizon^®^ Sextant™ Rod Insertion (n=54; 71.1%) or Longitude™ (n=22; 28.9%) Systems between August 2003 and January 2007. The indications for surgery were unstable type B fractures, Type A1 and A2 fractures with > 25% anterior height loss or additional disc injury (in need for combined anterior surgery), Type A1 fractures in obese and geriatric patients as well as burst fractures, Type A3 in patients younger than 40 years of age. Patients with verified neurological deficits were excluded from the study. Dorsal compression of spinal canal was treated using open surgery including laminectomy and consecutive conventional instrumentation. Patients with anterior compression without signs of posterior injury were operated using single anterior approach to clear the spinal canal. Conservative treatment was conducted in patients with stable spine injuries who showed sufficient compliance and will to wear external bracing. This report features followup data evaluation of 76 patients for a period of six months post surgery. The mean age was 53.3 years (SD ± 16.9). Geriatric patients (n=15) aged over 70 showed low preoperative physical fitness using the ASA score^12^ assigning seven patients to Class 2, six to Class 3 and two to Class 4 [Figure 1]. In the group of younger patients, 37 patients (48.7%) suffered from moderate to severe multiple injuries with an injury severity score of >16 which is indicated by high counts in ASA Class 4 and 5 [Figure 1]. Verification of fractures was performed using plain X-rays and additional MRI for discoligamentous injury or to differentiate old from acute osteoporotic fractures. Fifty patients (65.8%) suffered from single vertebral fractures, whereas in 26 cases (34.2%) multiple vertebral body fractures were undertaken for surgical stabilization. Fracture type definition was performed using the Magerl classification^1^ [Figure 2].

**Figure 3.1:**
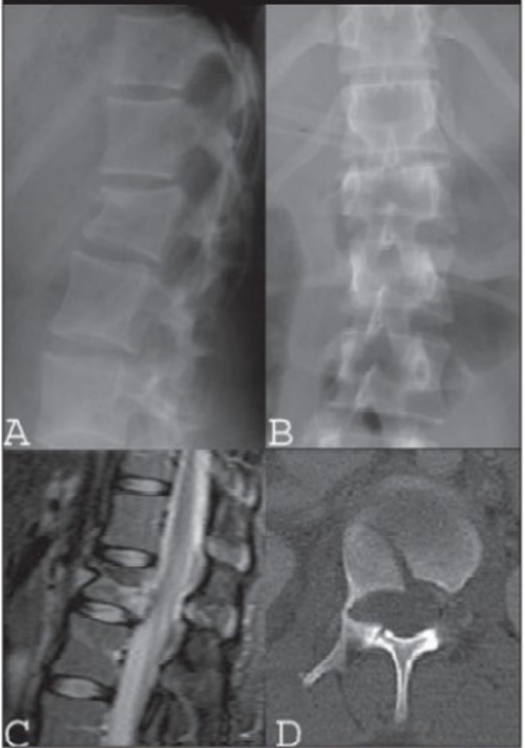
Lateral (A) and AP x-ray (B), mid sagittal T2WI of MRI (C) and CT scan (D) in a 16 years old female patient with flexion-distraction injury and rotatory instability following a horse riding accident

An operating team consisting of two experienced trauma and orthopedic surgeons performed all procedures. The surgical technique is described in detail elsewhere.^6^ The patients were positioned prone. C-arm fluoroscopy device was used for guidance of percutaneous screw placement, as described by Magerl *et al*. and modified by Wiesner *et al*.^13^ Stab incisions were made and transpedicular K-wires were placed. Using dilators, the soft tissue was bluntly separated. Cannulated pedicle screws were placed via the K-wires. The positioning of pedicle screws was controlled by using C-arm fluoroscopy. Pedicle screw extenders were used to define rod length and Sextant rod inserter was attached. Via additional cranial stab incisions the Sextant allowed percutaneous insertion of the rod in a curvilinear path connecting both screw head openings through additional stab incisions [Figure 3.1–3.3]. For the Longitude system, the rod was placed by a rod extender using free-hand technique. Single dorsal approach for spinal stabilization was done in 51 cases (67.1%) compared to dorso-ventral operations in 25 cases (32.9%) for discoligamentous injury of the vertebral disc or fracture-associated with spinal compromise in e.g. Type A3 fractures. In these combined procedures, anterior videoscopy-assisted left-sided retroperitoneal approach or left-sided thoracotomy was performed. For monosegmental fusion a discectomy and intervertebral allogenous bone graft was used. Bisegmental fusions were performed using a distractable cage following discectomy and corporectomy, supplemented by an anterior screw-rod implant (Antares™, Medtronic). Multi-level instrumentation using Longitude™ Rod Insertion System [Figure 4] was done in 16 out of 76 patients (21.0%).

**Figure 3.2:**
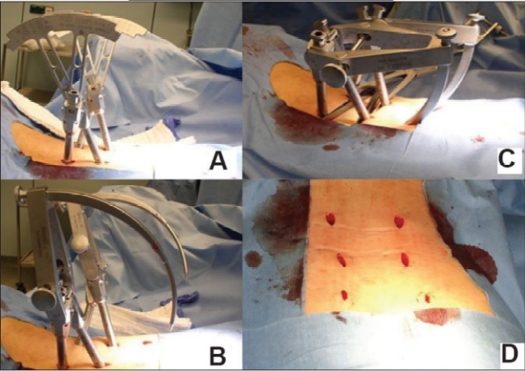
Intraoperative photographs show simultaneous insertion of two Sextant Fixators. Use of rod templates to determine the length of the rod (A). Inserted via stab incisions. Conventional rod distractors are used for added distraction force to the posterior wall fragment (B) and Rods are attached to the Sextant Introducer (C). Final approachrelated injury is minimal as demonstrated by these < 2 cm long stab incisions for a bisegmental internal fixator (D)

**Figure 3.3:**
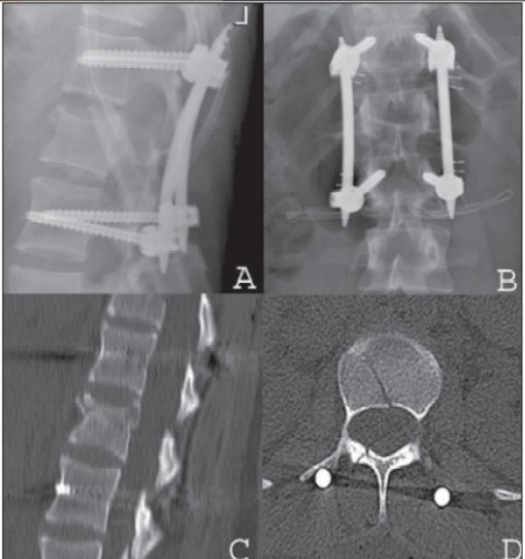
Post operative lateral (A) and AP X-ray (B), sagittal reconstruction CT (C) and axial CT (D) shows anatomic reduction and anterior height restoration. Uneventful recovery and percutaneous implant removal six months post trauma was performed

**Figure 4:**
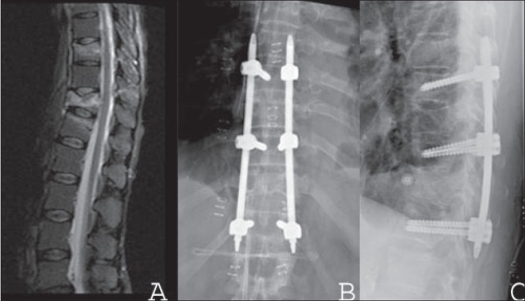
Mid sagittal T2WI (A) MRI shows flexion-distraction injury of the thoracolumbar spine in a 55 yrs old female with no neural deficit. X-ray of dorsal spine AP and lateral (B,C) shows stabilization with percutaneous longitude Longitude™ implant. Early ambulation without external bracing was achieved on day one post surgery. Wound healing was primary; no substantial complaints were reported on follow-up.

Related to the high number of Type A fractures, only six (7.9%) out of 76 patients demonstrated neurological deficit prior to surgery. One patient showed incomplete paresis and recovered following immediate dorso-ventral approach including spinal canal clearance. Most patients (n=72; 94.7%) complained about local fracture-associated pain and discomfort during preoperative physical examination.

Upon patient agreement, data were collected prospectively and followup investigations were performed eight weeks and six months post surgery. The presented clinical outcome parameters were recorded at the six-month time point. For evaluation of general health status, the short form 36 questionnaire (SF-36) was completed.

## RESULTS

Blood transfusions were required in three out of 76 patients (3.9%) in whom additional anterior approach, e.g. corpectomy was necessary.

**Figure 5:**
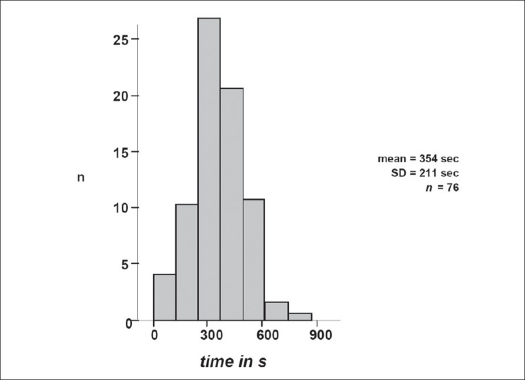
Duration of C-arm fluoroscopy for positioning of pedicle screws and intraoperative verification of correct implant positioning shows mean radiation time of 354 seconds (n = patient number)

In contrast to open surgery, where the surgeon controls correct screw placement by palpating the screw canal through the pedicle into the vertebral body, percutaneous placement of pedicle screws is associated with increased radiation time. Short-level dorsal fusion procedures (n = 60) revealed mean radiation time of 354 seconds (SD ± 211 sec) for percutaneous insertion of four pedicle screws, rod insertion and radiographic control of operative result prior to completion of surgery [Figure 5]. Mean duration of instrumentation procedure itself was 47.0 min (SD ± 14.4) for the whole study population of 76 patients. In selected cases, accomplishment of surgery was achieved in less than 45 min. In more than half (56.2%) of the patients, a minimum of 22.5 min and 36.5 min for Sextant™ or Longitude™, respectively was required.

**Figure 6:**
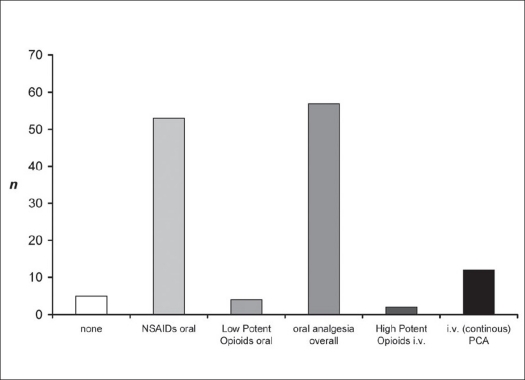
Different forms of analgesia in patients following percutaneous minimal-invasive dorsal fusion procedure. Low demand of analgesics in patients following use of minimal-invasive stabilization is demonstrated. Fifty-seven out of 76 patients were sufficiently treated by oral analgesia like NSAIDs, only. In 12 patients treated with additional surgery, initial patient-controlled intravenous analgesia was necessary for pain reduction

Overall analgesia demand was documented. Intravenous analgesia via a patient-controlled analgetic (PCA) device was set up using the opioid piritramide (Dipidolor^®^, Bolus 2-3 mg, max. 25-40 mg within 4 h). No patient with dorsal instrumentation alone was in need of PCA. Patients requiring PCA (n=12; 15.8%) had associated trauma or were subjected to additional e.g. anterior spine surgery. As demonstrated in Figure 6, the total need for analgesia following minimal-invasive dorsal instrumentation using either Sextant™ or Longitude™ System was low. The majority of patients (57 out of 76; 75.0%) were sufficiently treated by oral analgesics only, in which non-steroidal anti-inflammatory drugs (NSAIDs) like ibuprofen and etoricoxib were used as single medication in 53 patients (69.7%). Only two geriatric patients (2.6%) required high potent opioids like oxycodon or fentanyl frequently, which might be ascribed to their previous transdermal opioid medication.

The hospital stay was calculated from day of surgery to discharge from the hospital. The mean hospital duration was 18.7 days (SD ± 8.8). The mean hospital discharge was accomplished within 10.2 days, omitting those patients that were transferred back intraday to their primary departments, e.g. oncology or intensive care. Postoperative complications have been documented in three (3.9%) out of 76 cases. One paravertebral hematoma needed a surgical revision. One patient complained of persisting skin irritation which was attributed to an insufficient suture of the fascia and had to be revised. One implant failure was seen in a breakage of a cranial pedicle screw of a bisegmental fixator (stand alone, no anterior fusion) in a young patient at followup at six months. No problems with wound healing e.g. swelling, seroma formation or paravertebral hardness were observed. Polytraumatized patients with blunt chest trauma (n=20) developed Acute Respiratory Distress Syndrome (ARDS) in two cases and overall rate of septic complications has been found in three out of 27 patients suffering from multiple injuries. None of these were associated with posterior surgery-related complications like implant infection or revision surgery. Radiographic monitoring at six months revealed no significant loss of retention or implant failure, necessitating operative revision. These findings are in line with studies of conventional dorsal fixation procedures.

On followup examination the patients had to respond to the SF-36 general health questionnaire. Thirty-two patients (42.1%) had no substantial discomfort and pain as compared to the time before surgery. Six months following surgery, 58 (76.3%) met their expectations or were highly pleased by their individual postoperative results.

## DISCUSSION

Conventional, open dorsal instrumentation of the thoracolumbar spine following trauma has been performed for more than 30 years. This approach requires extensive tissue dissection to expose the bony structures of the spine and for pedicle screw fixation, to provide enough space for lateral-to-medial orientation for optimum screw placement. Consecutively, paravertebral muscles are denervated and dissection leads to muscle and soft tissue ischemia potentially contributing to some cases of failed fracture stabilization.^14^ Open approach for simple laminectomy can lead to atrophy of the posterior paraspinous muscles and a poor clinical result.^15^ Following a posterior lumbar fusion the patients are not capable of increasing their muscle strength even under an intensive exercise program, most likely due to approach-related denervation and muscle injury.^16^,^17^ For polytraumatized or septic patients suffering from critical illness neuropathy and catabolism state this process might be even aggravated. It was also shown that physical compression by soft tissue retractors during surgery induces time-dependent muscular histological damage via increased intramuscular pressure.^18^–^23^ Furthermore, conventional approach to the spine is associated with extensive blood loss, risk of wound infection and prolonged hospitalization.^2^,^24^

The minimally invasive percutaneous stabilization of the spine might be the right concept to minimize such approach-related morbidity and secondary iatrogenic soft tissue trauma.^20^,^25^ Early percutaneous fixation in our predominantly trauma-associated spine patients enables earlier mobilization, especially for ICU and geriatric patients which might contribute to improved outcome regarding pulmonary or thromboembolic complications, and even decubitus ulcers.^9^,^10^,^25^–^28^ None of our polytraumatized patients developed secondary complications due to minimal-invasive posterior instrumentation. Furthermore a low overall rate for ARDS and sepsis might be attributed to quick surgery and low antigen load. In addition, local wound infection (0 out of 76) and implant failure (1 out of 76) rates seem to be lower or in the range of reports using conventional posterior instrumentation.^29^–^31^

Kim *et al.* enrolled 19 patients in a prospective study to evaluate the morbidities related to minimally invasive spinal surgery. He observed less paraspinal muscle damage in percutaneous pedicle screw fixation techniques compared to open pedicle screw fixation to support the positive effects on postoperative trunk muscle performance.^32^ Assaker reported (n=40) exceptionally good results considering implant behavior and patient outcome during a mean followup of 12 months in patients suffering from A and B type fractures.^33^ Wild *et al.* reported (n=21) on consecutive non-randomized patients with thoracolumbar vertebral body fractures without neurological symptoms, which had been stabilized without any intervertebral body fusion and were examined retrospectively more than five years after trauma. He reported significantly lower blood loss in minimally invasive surgery but a similar operating time; X-ray exposure time and the loss of correction were identical in the minimally invasive and conventionally operated group.^34^ Hence he did not favor the minimal-invasive approach. In line with the author's findings, we reported no posterior instrumentation-related blood loss and elevated X-ray exposure during surgery, which is caused by a more difficult approach to define the screw entry points, and correct trajectory in the totally percutaneous procedure. Nevertheless, we found mean operating time (47.0 min ± 14.4) to be lower in the minimal-invasive approach than the conventional pedicle screw instrumentation ranging from 81 min^34^ to 240 min.^35^

Although our outcome shows encouraging results and with easy intraoperative handling of the sophisticated implant, some drawbacks have been detected. The minimal-invasive approach does not allow placement of cross-links, which would be the precondition for stabilization of longer-ranging and seriously unstable segments. In comparison to fixed/ Schanz-screw implants, the system has limited capability for closed reduction. Although compression handles allow for distraction and compression of the instrumented segment, the polyaxial screw design directs compression/distraction forces to the posterior column, only. Therefore excessive reposition maneuvers are not feasible and sufficient reduction of the fracture should be achieved using optimized posture and manual reduction including e.g. axial leg tension or direct sagittal manipulation of the injured segment. In case of improper reduction, open surgery should be performed using rigid, fixed angle screws. The perspective of a minimal-invasive procedure should not divert the surgeon from the fact that an anatomically correct reduction is the key point of surgery.

Various percutaneous applied internal fixators have been described^4^,^36^–^40^ but currently none is specifically designed for trauma. Research cohorts remain small in number and longterm followup studies are lacking.^5^,^6^,^41^,^42^ Our preliminary data resemble one of the largest cohorts in minimal-invasive spine fixation in trauma patients. The results show favorable outcomes in patients through shortened operative time, limited soft tissue damage and quick mobilization following surgery. However, these data describe first experiences in the use of minimal-invasive dorsal instrumentation implants and the study design limits scientific value. The indications and implants are currently in evolution. Further randomized prospective controlled trials should assess the advantages and disadvantages of the initial encouraging results.
